# Supplementary material for: Biochemical mechanisms determine the functional compatibility of heterologous genes
Source: Nat Commun. 2018 Feb 6;9:522. doi: 10.1038/s41467-018-02944-3 (PMC5802803; doi:10.1038/s41467-018-02944-3)
Supplement: Supplementary file 1 — Supplementary Information [file 41467_2018_2944_MOESM1_ESM.pdf]

Supplementary Information

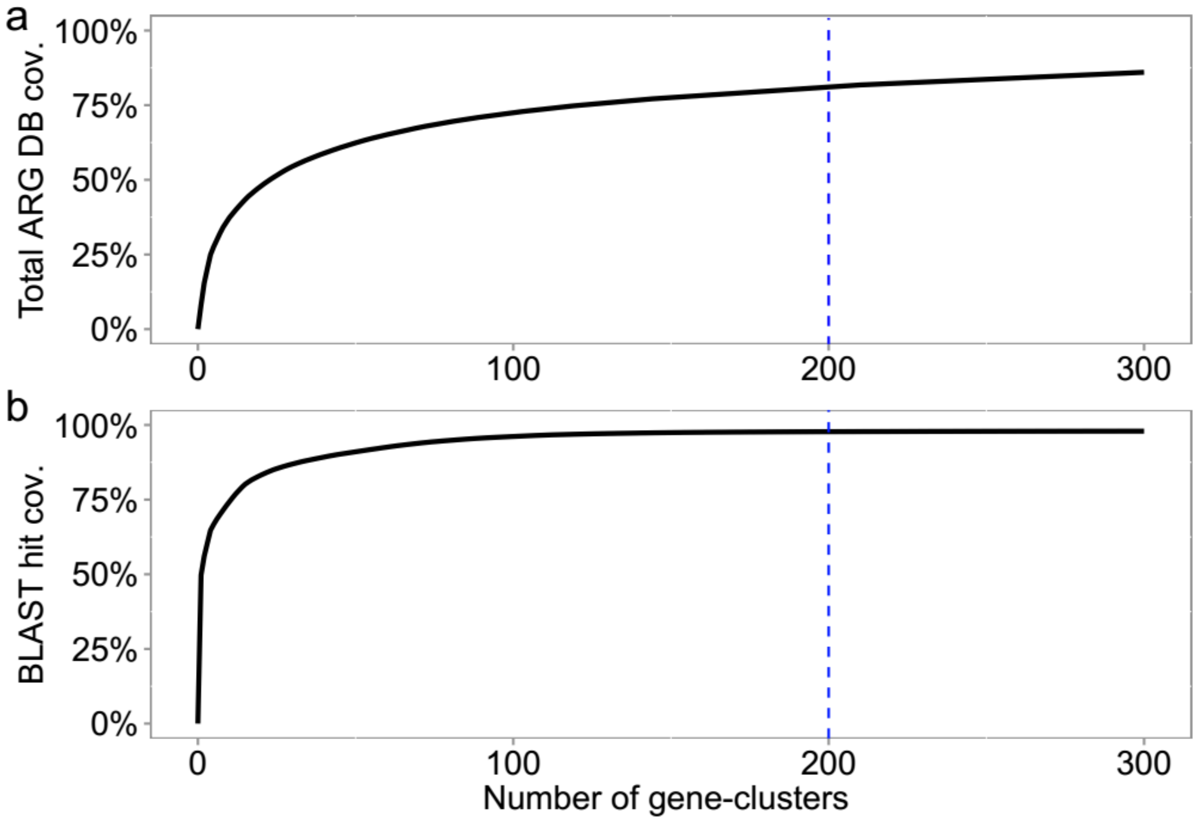

Supplementary Figure 1:

(**a**) Rarefaction plots depicting the cumulative coverage of the included resistance gene databases by the included ARG clusters (**b**) Cumulative coverage of NCBI's NT and Genomes sequence databases as a function of added clusters. The 200 included clusters are indicated by the blue dotted line.

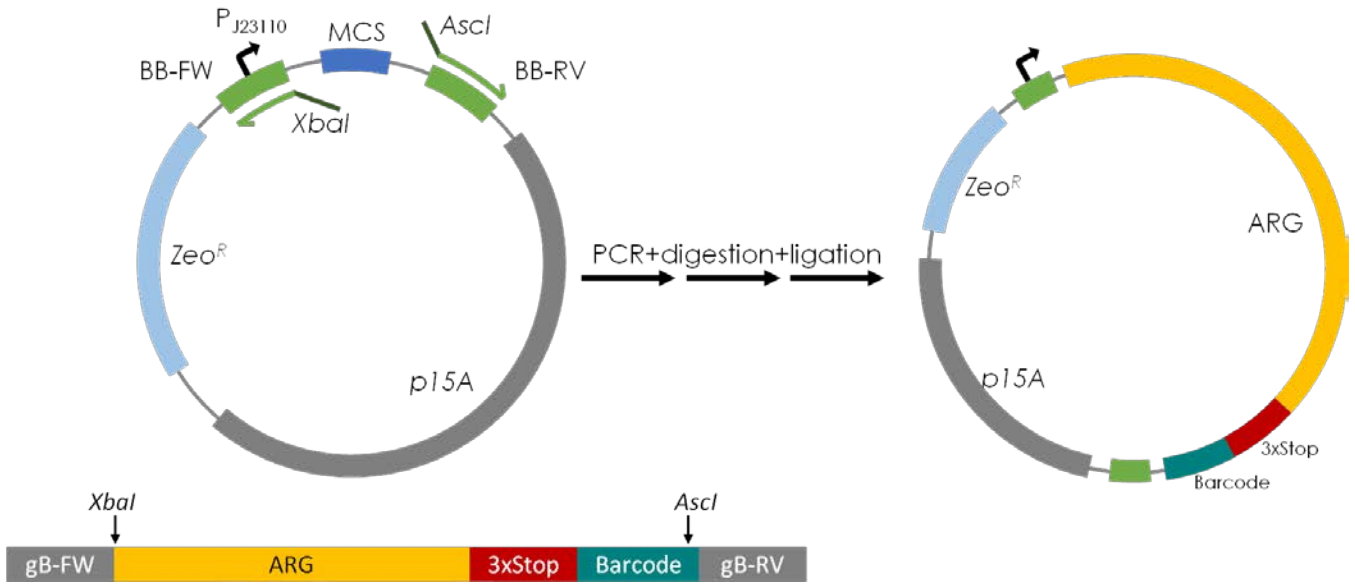

Supplementary Figure 2:

Cloning procedure. The vector backbone was amplified with restriction sites and digested by Xbal and AscI to allow ligation of the final library construct. The ordered genes were designed with primer binding sides (gB-FW and gB-RV) to allow amplification. 3 stop codons were inserted downstream each cloned gene to ensure proper translational termination and a 7nt barcode was incorporated for easy sequence validation.

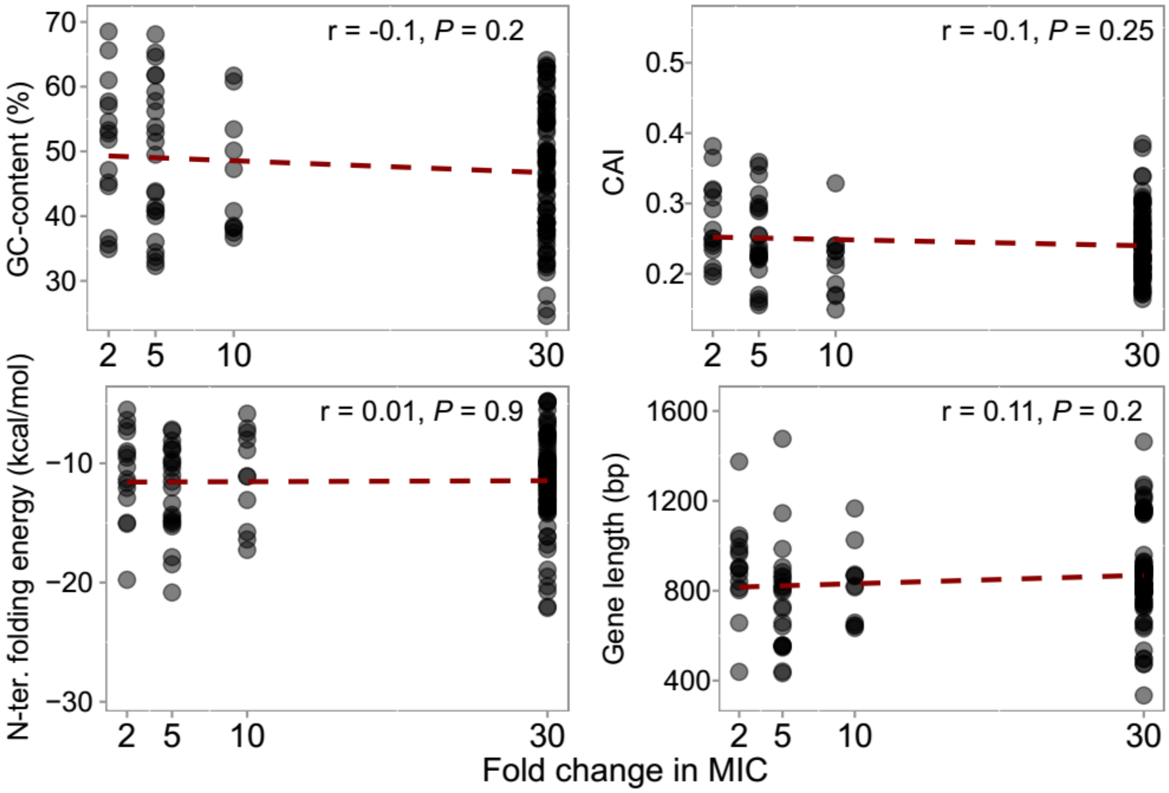

Supplementary Figure 3

Correlations of sequence parameters and resistance level for functional resistance genes (n = 126). The Spearman correlation coefficients are shown.

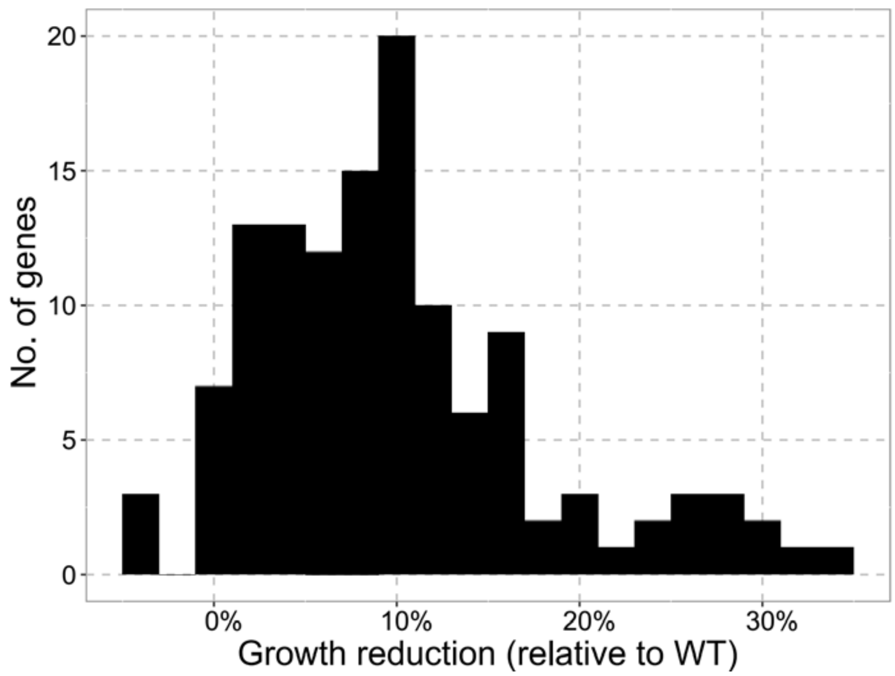

Supplementary Figure 4

Growth rate distribution of all 126 functional genes tested. The average of at least 3 replicates is shown.

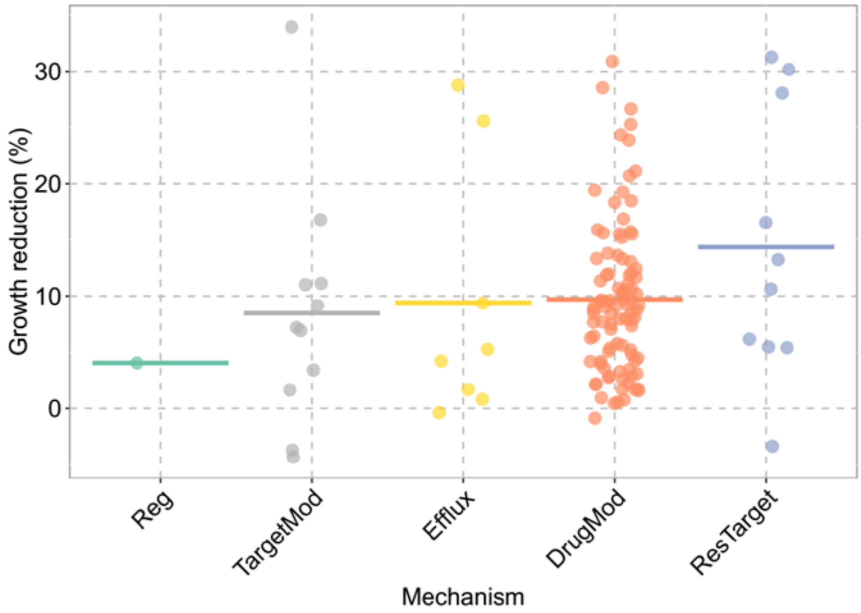

Supplementary Figure 5

The impact on the growth rate imposed by ARGs on *E. coli* stratified on resistance mechanism. The average value measured for at least 3 biological replicates is shown.

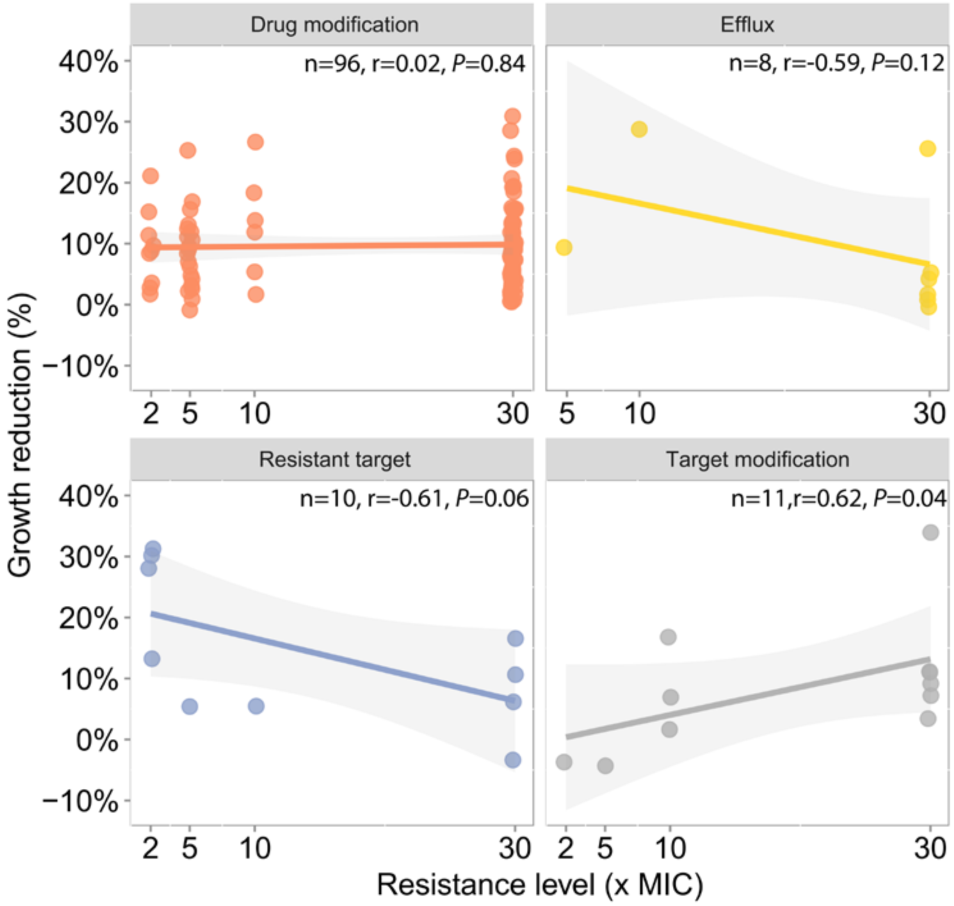

Supplementary Figure 6

Correlation between MIC and growth rates for the different mechanistic classes. Regulators were omitted due to the small sample size (n=1; see above). The average value measured for at least 3 biological replicates is shown. The shading represents the standard-error of the linear fit and the Spearman correlation coefficients are shown.

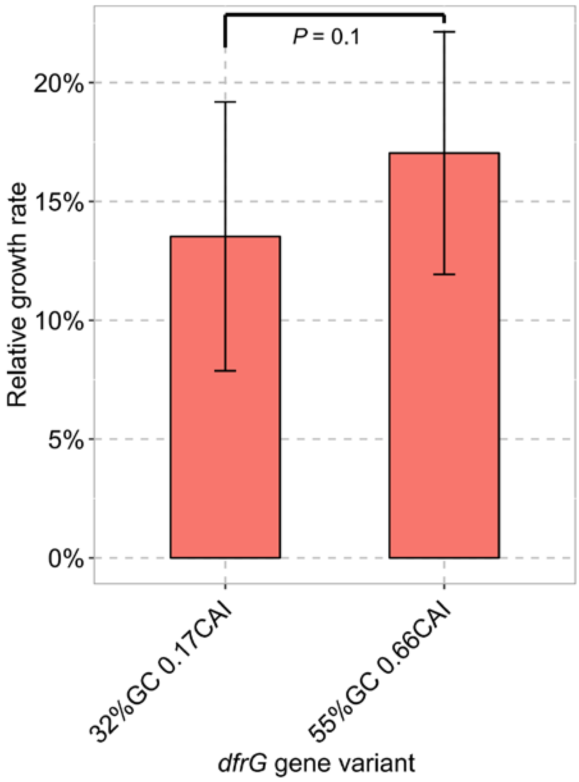

Supplementary Figure 7

A codon optimized *dfrG* gene variant was synthesized to test the influence of gene composition on growth rate in *E. coli*.

Optimized gene sequence:

”<u>ATGAAAGTTTCTTTGATTGCTGCGATGGAT</u>AAAAACCGCGTGATCGGCAAAGAGAACGACATCCCGTGGCGCATCCCGAAA
GACTGGGAGTACGTGAAAAACACCACCAAAGGCCACCCGATCATCCTGGGCCGCAAAAACCTGGAGAGCATCGGCCGCGCG
CTGCCGGACCGCCGCAACATCATCCTGACCCGCGACAAAGGCTTCACCTTCAACGGCTGCGAGATCGTGCACAGCATCGAGG
ACGTGTTCGAGCTGTGCAAGAACGAGGAGGAGATCTTCATCTTCGGCGGCGAGCAGATCTACAACCTGTTCTTCCCGTACGT
GGAGAAGATGTACATCACCAAAATCCACCACGAGTTCGAGGGCGACACCTTCTTCCCGGAGGTGAACTACGAGGAGTGGAA
CGAGGTGTTCGCGCAGAAAGGCATCAAGAACGACAAAAACCCGTACAACTACTACTTCCACGTGTACGAGCGCAAGAACCT
GCTGAGCTGA”

The first 10 codons were kept unchanged to avoid changes in mRNA folding from affecting expression. The growth rate was measured for the optimized (55% GC and 0.68 CAI) and the wild type (32% GC and 0.17 CAI) variant. No significant difference in growth was observed (Mann-Whitney U-test, P = 0.1). Error-bars show the standard deviation (SD) of 16 biological replicates.

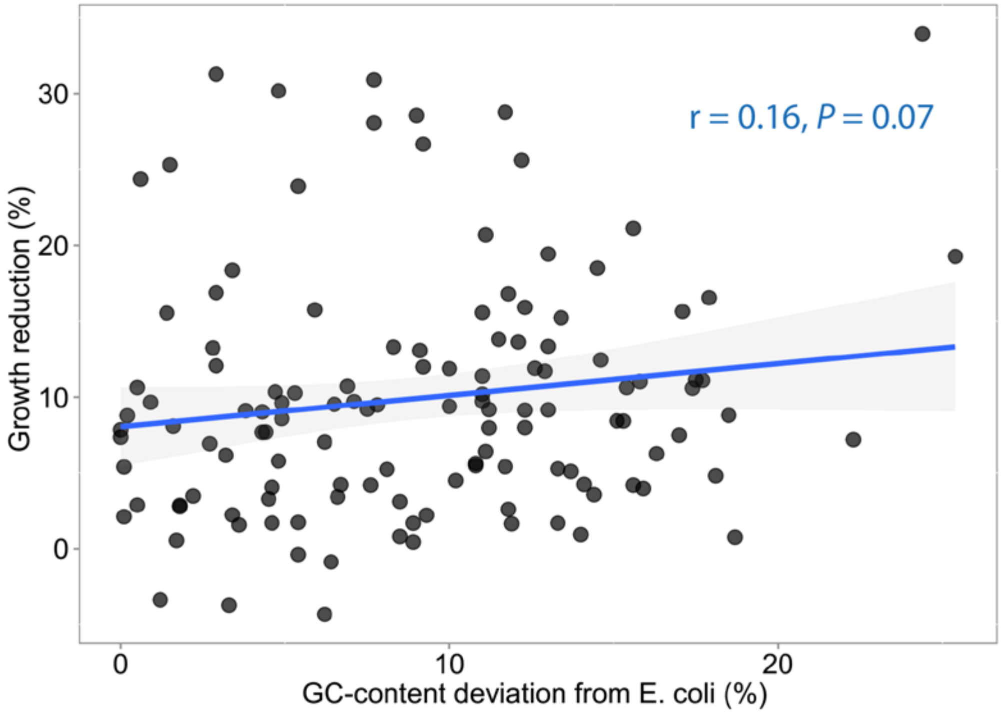

Supplementary Figure 8

Correlation between relative GC-content and growth rate for all functional genes (n = 126). The average value measured for at least 3 biological replicates is shown for each gene. The shading represents the standard-error of the linear fit and the Spearman correlation coefficients are shown.

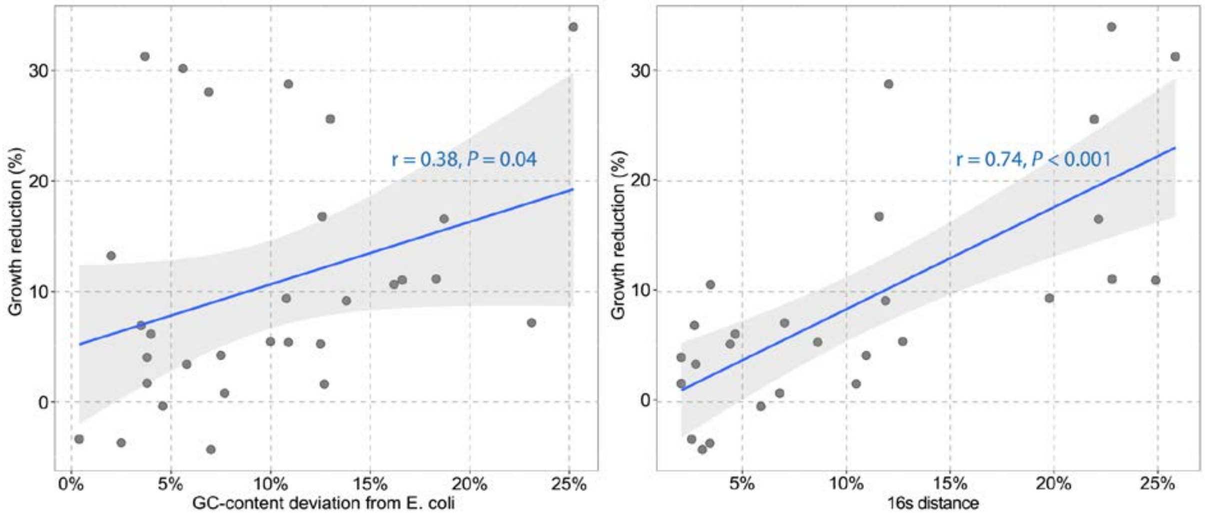

Supplementary Figure 9

Correlation of growth rate with GC-content and 16s distance for cell-interacting genes (n = 27). The average value measured for at least 3 biological replicates is shown for each gene. The shading represents the standard-error of the linear fit and the Spearman correlation coefficients are shown.

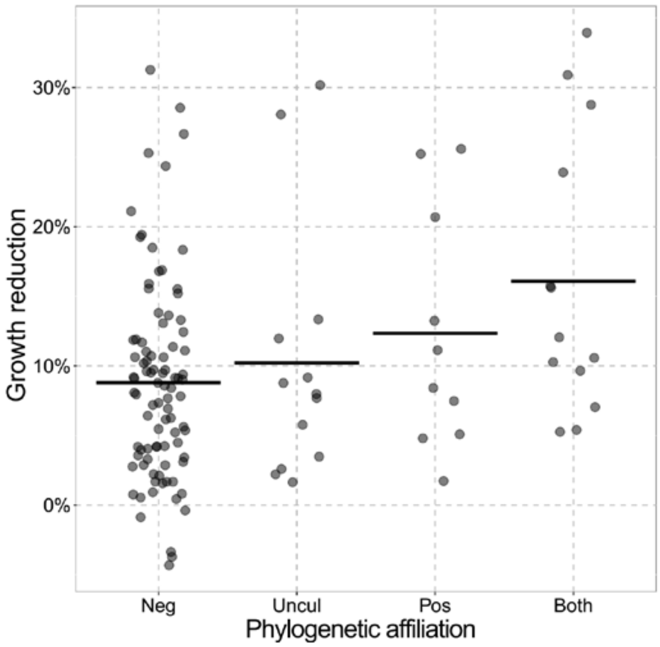

Supplementary Figure 10

The impact on the growth rate of *E. coli* stratified on phylogenetic origin at the Gram-class affiliation for all functional genes (n = 126). Gram-negative organisms: Neg (n = 90); Gram-positive organisms: Pos (n = 10); both Gram-negative and Gram-positive organisms: Both (n = 14); or none of currently sequenced genomes in RefSeq: Uncul(n = 12). The average value of least 3 biological replicates is shown.

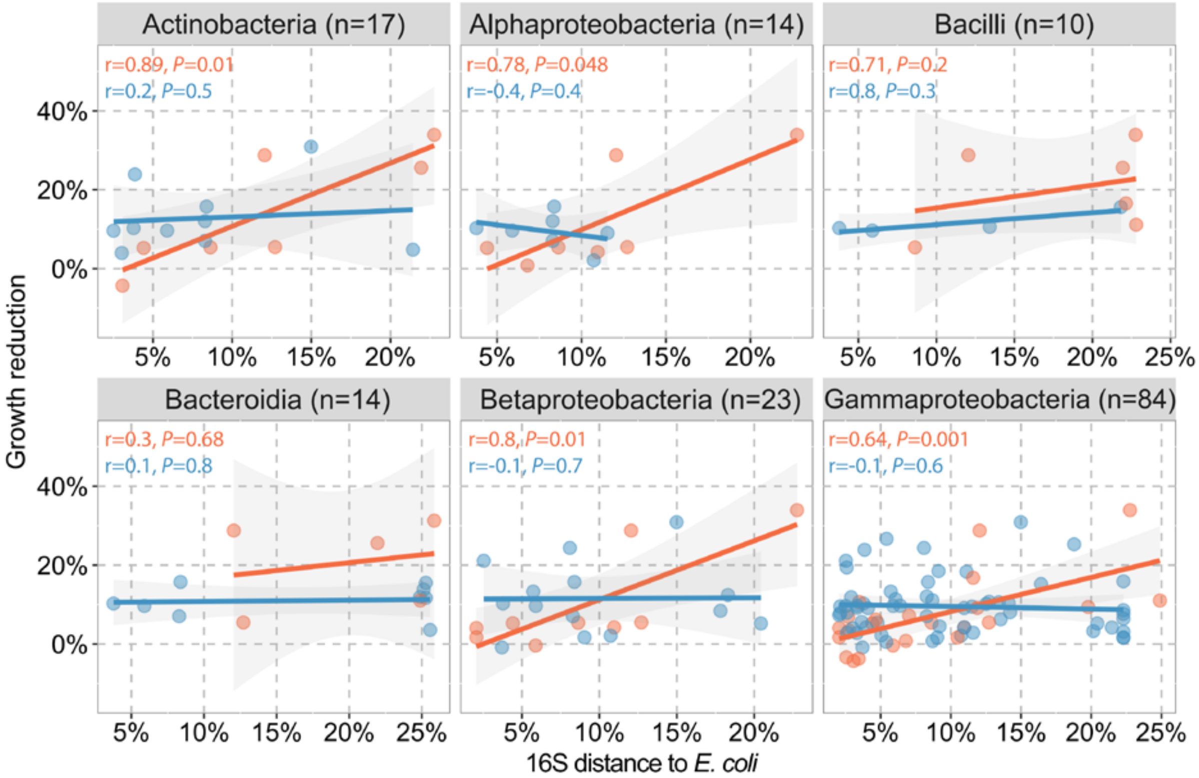

Supplementary Figure 11

The effect of the average 16S distance on the growth reduction imposed by drug-interacting (blue) and cell-interacting (orange) genes detected in different phylogenetic classes. Only taxonomical classes with more than two genes in each category were included. There was no significant difference in the correlations observed between phylogenetic class affiliations in a multiple linear regression model including these factors (ANOVA, $P = 0.23$). The average value measured for at least 3 biological replicates is shown. The shading represents the standard-error of the linear fit and the Spearman correlation coefficients are shown.

Supplementary Table 1 – MG1655/pZAT MICs:

Antibiotic	MIC (µg/ml)
Amikacin	8
Amoxicillin	8
Aztreonam	0.1
Cefuroxime	4
Chloramphenicol	4
Ciprofloxacin	0.0075
Clindamycin	26.67
Colistin	0.33
D-cycloserine	80
Erythromycin	16
Fosfomycin	0.42
Gentamicin	2
Mecillinam	0.16
Meropenem	0.12
Nitrofurantoin	16
Sulfamethoxazole	128
Streptomycin	4
Tetracycline	1
Trimethoprim	0.25
Vancomycin	128

Supplementary Table 2 - GLM:

Sequential fitting of a logistic regression model with absolute functionality in *E. coli* as dependent variable. A model was fitted using the *glm* function in R. Each expanded model was assessed as a chi-squared statistics of differences in deviance/error compared to the previous model (*P*-value added factor) and the null-model (*P*-value all). dGmRNA: N-terminal mRNA folding energy; GramClass: whether the gene is affiliated with Gram-positives only, Gram-negatives only or Both, when blasted against RefSeq.

**Model components**	**Deviance**	**DF**	***P*-value added factor**	***P*-value all**
null model	263.58	199	NA	NA
GC	260.87	198	0.09	0.09
GC+CAI	255.8	197	0.025	0.02
GC+CAI+dGmRNA	254.96	196	0.36	0.034
GC+CAI+dGmRNA+Mechanism	190.99	192	4.24E-13	4.42E-13
GC+CAI+dGmRNA+Mechanism +DrugClass	150.98	180	7.16E-05	2.66E-15
GC+CAI+dGmRNA+Mechanism +DrugClass+GramClass	146.56	177	0.21	6.11E-15

Supplementary Table 3 – MLR

Sequential fitting of a multiple linear regression model to the change in growth rate imposed by functional resistance genes in *E. coli*. A model was fitted using the *lm* function in R. Each expanded model was assessed by the F-statistics of explained variance compared to the model without the added factor (*P*-value added factor) and the null-model (*P*-value all). dGmRNA: N-terminal mRNA folding energy; GramClass: whether the gene is affiliated with Gram-positives only, Gram-negatives only or Both, when blasted against RefSeq.

**Model components**	**Adj. R^2**	**DF**	***P*-value added factor**	***P*-value all**
GC	-0.002	124	0.4	0.4
GC+CAI	-0.002	123	0.3	0.4
GC+CAI+dGmRNA	-0.007	122	0.5	0.5
GC+CAI+dGmRNA+GeneLength	-0.01	121	0.58	0.6
GC+CAI+dGmRNA+GeneLength +DrugClass	0.16	111	3.00E-04	1.00E-03
GC+CAI+dGmRNA+GeneLength+DrugClass +GramClass	0.2	108	0.038	4.00E-04
GC+CAI+dGmRNA+Mechanism+DrugClass +GramClass+Mechanism	0.21	105	0.34	6.00E-04
GC+CAI+dGmRNA+Mechanism+DrugClass +GramClass+Mechanism+MaxMIC	0.2	104	0.59	0.001

Supplementary Table 4 – Native *E. coli* genes

Due to their high abundance in resistance gene databases, a number of native *E. coli* genes vaguely associated with antibiotic resistance were included in the synthetic gene selection. For the full list of gene information, see Supplementary Data 1.

Cluster	GeneName	Mechanism	Drug class	GC (%)	CAI
77	*pmrC*	Reg	Unk	49.5	0.315
89	*emrB*	Efflux	Mul	56.2	0.279
104	*mdtP*	Efflux	Mul	55.8	0.279
108	*phoQ*	Reg	Mul	51.3	0.25
121	*mdtD*	Efflux	Mul	55.5	0.271
124	*baeS*	Reg	Unk	53.7	0.319
136	*hmrM*	Efflux	Mul	53	0.331
157	*mdtA*	Efflux	Unk	55.3	0.353
164	*mdtM*	Efflux	Mul	53.8	0.266
172	*mdtG*	Efflux	Mul	52.5	0.222
184	*MdtH*	Efflux	Mul	55.3	0.294
213	*mdtL*	Efflux	Mul	53.1	0.252
216	*EmrA*	Efflux	Mul	53.4	0.386
257	*BasS/PmrB*	Reg	Pol	54.2	0.256
292	*mdtN*	Efflux	Mul	55.9	0.349
326	*arnC*	TargetMod	Pol	49.8	0.3
791	*HNS*	Reg	Unk	46.4	0.56
228	*AcrE*	Efflux	Mul	52.1	0.32
221	*mdtA*	Efflux	Unk	55.3	0.353
135	*cpxA*	Reg	Ami	54.6	0.365
