# Supplementary material for: Biochemical mechanisms determine the functional compatibility of heterologous genes
Source: Nat Commun. 2018 Feb 6;9:522. doi: 10.1038/s41467-018-02944-3 (PMC5802803; doi:10.1038/s41467-018-02944-3)
Supplement: Supplementary file 3 — Description of Additional Supplementary Files [file 41467_2018_2944_MOESM3_ESM.pdf]

Description of Additional Supplementary Files

File Name: Supplementary Data 1
Description: Table containing sequences and information on all synthetic genes included in the study.
